# A preliminary study of individual cognitive behavior therapy for social anxiety disorder in Japanese clinical settings: a single-arm, uncontrolled trial

**DOI:** 10.1186/1756-0500-6-74

**Published:** 2013-02-28

**Authors:** Naoki Yoshinaga, Fumiyo Ohshima, Satoshi Matsuki, Mari Tanaka, Tomomi Kobayashi, Hanae Ibuki, Kenichi Asano, Osamu Kobori, Tetsuya Shiraishi, Emi Ito, Michiko Nakazato, Akiko Nakagawa, Masaomi Iyo, Eiji Shimizu

**Affiliations:** 1 Department of Cognitive Behavioral Physiology, Chiba University Graduate School of Medicine, 1-8-1 Inohana, Chuo-ku, Chiba, 260–8670, Japan; 2 Research Center for Child Mental Development, Chiba University Graduate School of Medicine, 1-8-1 Inohana, Chuo-ku, Chiba, 260-8670, Japan; 3 Center for Forensic Mental Health, Chiba University, 1-8-1 Inohana, Chuo-ku, Chiba, 260-8670, Japan; 4 Department of Psychiatry, Chiba University Graduate School of Medicine, Chiba, 1-8-1 Inohana, Chuo-ku, 260-8670, Japan

**Keywords:** Cognitive behavioral therapy, CBT, Social anxiety disorder, Social phobia, SAD, Japanese

## Abstract

**Background:**

Cognitive behavior therapy (CBT) is regarded as an effective treatment for social anxiety disorder (SAD) in Europe and North America. Individual CBT might be acceptable and effective for patients with SAD even in non-Western cultures; therefore, we conducted a feasibility study of individual CBT for SAD in Japanese clinical settings. We also examined the baseline predictors of outcomes associated with receiving CBT.

**Methods:**

This single-arm trial employed a 14-week individual CBT intervention. The primary outcome was the self-rated Liebowitz Social Anxiety Scale, with secondary measurements of other social anxiety and depressive severity. Assessments were conducted at baseline, after a waiting period before CBT, during CBT, and after CBT.

**Results:**

Of the 19 subjects screened, 15 were eligible for the study and completed the outcome measures at all assessment points. Receiving CBT led to significant improvements in primary and secondary SAD severity (*p*s < .001). The mean total score on the Liebowitz Social Anxiety Scale improved from 91.8 to 51.7 (before CBT to after CBT), and the within-group effect size at the end-point assessment was large (Cohen’s *d* = 1.71). After CBT, 73% of participants were judged to be treatment responders, and 40% met the criteria for remission. We found no significant baseline predictors of those outcomes.

**Conclusion:**

Despite several limitations, our treatment—which comprises a 14-week, individual CBT program—seems feasible and may achieve favorable treatment outcomes for SAD in Japanese clinical settings. Further controlled trials are required in order to address the limitations of this study.

**Trial registration:**

UMIN-CTR UMIN000005897

## Background

Social anxiety disorder (SAD; also known as social phobia) is characterized by fear of social situations involving performance or interaction [1]. SAD is one of the most prevalent psychiatric disorders in developed and developing countries [2] and is associated with substantial comorbidity (like many other anxiety and depressive disorders), functional disability (including social and occupational impairment), low health-related quality of life, and economic burden [3-5].

Pharmacotherapy and psychotherapy have been recommended as the first-line treatments for SAD [6-8]. In terms of pharmacotherapy, a growing database of randomized, controlled trials demonstrates that selective serotonin reuptake inhibitors (SSRIs) are effective and well tolerated [8]. In psychotherapy, cognitive behavioral therapy (CBT) has consistently been shown to be effective in randomized, controlled trials [9]. While no clear evidence has shown that the combination of SSRIs and CBT is more effective than single-modality treatment [10,11], CBT has a number of potential advantages over pharmacotherapy in the treatment of anxiety disorder: longer effects, fewer adverse effects, smaller relapse rates, and greater acceptability [12-14]. Pharmacotherapy has disadvantages such as more side effects and higher rates of relapse with the discontinuation of medication [15,16].

CBT was introduced into Japanese psychiatry in the late 1980s, and awareness of the effectiveness of CBT has spread, not only among professionals and academics but also among the general public. In April 2010, CBT for mood disorders (beyond that for anxiety disorders) began to be covered by Japan’s national health insurance system. Nevertheless, a recent nationwide survey in Japan demonstrated that only 28% of medical facilities reported being able to conduct any form of psychotherapy satisfactorily [17] because of the limited availability of specialized practitioners. Only SSRIs (fluvoxamine and paroxetine) have been established as first-line treatments for Japanese patients with SAD as of 2012. It is therefore necessary to investigate whether CBT can achieve favorable treatment outcomes in Japanese SAD patients.

Previous reports about the effectiveness of CBT mostly came from Europe and North America, and CBT models and treatment components were developed in Western cultures with theoretical orientations typically constrained by Western conceptualizations of SAD. Cultural factors may be especially relevant to SAD pathology. For example, *taijin-kyofu-sho* (in Japanese, *taijin* means “interpersonal,” *kyofu* means “fear,” and *sho* means “syndrome”), which is listed in the appendix to DSM-IV, is said to be a culture-bound syndrome that is unique to East Asia. Although fear of interpersonal relations has been considered a culture-bound syndrome [18-20], it can also be classified under existing categories in the DSM-IV-TR [21-23]. The notion that fear of interpersonal relations is purely a culture-bound syndrome does not always hold true. Despite differences between the conceptualizations of SAD and *taijin-kyofu-sho*, patients suffering from SAD in different parts of the world share many features in common, and similar assessments and treatments have been utilized across the world [24].

Only Chen and colleagues [25-27] showed that group CBT can bring about a similar degree of symptom reduction for Japanese patients as for Western patients with SAD. However, no study has tested the effectiveness of individual CBT for SAD in Japan. It is necessary to investigate whether individual CBT can achieve favorable treatment outcomes in Japanese patients with SAD, because some recent studies from Europe and North America have suggested that individual CBT is more effective than group CBT [28,29]. In addition, SAD has commonly been found to be highly comorbid with other Axis-I disorders, such as depression, bipolar disorder, and other anxiety disorders. Therefore, it is also important to understand how comorbidity and other clinical demographics affect treatment outcomes for SAD in clinical settings.

Thus, the purposes of this study are to report the preliminary outcomes of an individual CBT program for SAD in Japanese clinical settings and to examine the baseline predictors of the short-term outcomes associated with receiving CBT. The hypothesis is that individual CBT will be associated with decreased SAD severity in Japanese clinical settings and achieve comparable effectiveness to applications reported in Western settings.

## Methods

### Participants

The criteria for inclusion in this study were a primary diagnosis of SAD according to the DSM-IV, age of 18–65 years, and at least moderately severe SAD (on the basis of a Liebowitz Social Anxiety Scale [LSAS] score ≥ 50) [30,31]. Because Sugawara et al. (2012) reported that the mean total LSAS score was 42.4 (average SD = 27.5) in healthy Japanese community-dwelling subjects (*N* = 929) [32], we set a cutoff score of 50 on the LSAS for screening patients as suffering from moderate–severe symptoms of SAD. So that the study population would reflect routine clinical practice, comorbid diagnoses were permitted if clearly secondary (i.e., the SAD symptoms were both the most severe and the most impairing).

The exclusion criteria were psychosis, pervasive developmental disorders/mental retardation, autism spectrum disorders (Autism Spectrum Quotient ≥ 32) [33], current high risk of suicide, substance abuse or dependence in the past 6 months, antisocial personality disorder, unstable medical condition, pregnancy, or lactation.

All patients were evaluated by a psychiatrist using the Structured Clinical Interview for Axis I Disorders (SCID-I) [34]. All patients were also screened for autism spectrum disorder with the Autism Spectrum Quotient [33] and the avoidant personality disorder section of the SCID–II [35], because those measures show some overlap with social-anxiety features and cannot be screened using SCID-I. Treatment history was confirmed by the prescribing clinician and by chart review.

### Interventions

The CBT intervention was conducted in 14 weekly 90-minute sessions. Because the CBT model developed by Clark and Wells [36] has shown excellent treatment outcomes [28,29,37-39], our CBT program is based on the model of Clark and Wells. The main steps in treatment were as follows The main steps in treatment were as follows: (a) developing an individualized version of the cognitive-behavioral model of SAD; (b) conducting role-play–based behavioral experiments with and without safety behaviors; (c) restructuring distorted self-imagery using videotape feedback; (d) practicing external focus and shifting attention; (e) behavioral experiments to test negative beliefs; (f) modifying problematic pre- and post-event processing; (g) discussing the differences between self-beliefs and other people’s beliefs (reflected in survey results); (h) dealing with the remaining assumptions (schema work); (i) rescripting early memories linked to negative images in social situations; and (g) preventing relapse. Homework was assigned after every session.

### Therapist and quality control

The CBT intervention was delivered by 6 therapists (3 clinical psychologists, 1 nurse, 1 psychiatrist, and 1 psychiatric social worker) who were experienced in the use of CBT for anxiety disorders and had completed the CBT training program at Chiba University (Chiba Improving Access for Psychological Therapies project). To check adherence to the protocol and assist with planning future sessions for each treatment, all therapists attended weekly group supervision sessions with other therapists and supervision sessions with a senior supervisor (ES). The senior supervisor also checked the quality of their CBT on the basis of the Cognitive Therapy Scale-Revised [40].

### Outcomes

The primary outcome measure was self-reported symptoms of social anxiety, as measured on the LSAS [30], which is the most frequently used scale for the assessment of SAD. To assure comparability with previous CBT studies using the model of Clark and Wells, patients also completed additional self-report measures of SAD severity: the Social Phobia Scale/Social Interaction Anxiety Scale (SPS/SIAS) [41], the Fear of Negative Evaluation Scale (FNE) [42], and the Fear Questionnaire – Social Phobia subscale (FQ-SP) [43]. Good reliability and validity of the Japanese versions have been reported for the LSAS, SPS, SIAS, and FNE [44-46].

### Study design

This study was conducted as a single-arm, open trial to report the preliminary outcomes and the feasibility of a CBT intervention for SAD in Japanese clinical settings. Because this study was the first trial to employ an individual CBT intervention for SAD in East Asia (particularly in Japan), a single-arm trial examining baseline predictors rather than an efficacy trial is an appropriate design [47].

After enrolling in the study, patients were placed on a 2-week waiting period to establish the baseline stability of their symptoms. At the end of the waiting period, the patients received a CBT intervention for 14 weeks. Concomitant medications were permitted if the dose had been stable for at least 4 weeks prior to study entry and remained stable throughout the study. Assessments were conducted at baseline (week 0), pre-CBT (before session 1/week 2), mid-CBT (after session 7/week 9), and post-CBT (after session 14/week 16) time points.

This study was conducted at the psychiatric outpatient section at Chiba University Hospital and was performed in compliance with the Helsinki Declaration. The study protocol was approved by the Ethics Committee of the Chiba University Graduate School of Medicine (Reference number: 1216) and was registered in the national UMIN Clinical Trials Registry (ID: UMIN000005897).

### Statistical analysis

The analysis was by intention-to-treat, and the last obtained data points for non-completers (because of adverse events, lack of compliance, etc.) were carried forward until the endpoint assessment. All statistical tests were two-tailed, and an α of .05 was employed. All of the data were analyzed using IBM SPSS Statistics for Windows, Version 20.0 (IBM, Armonk, New York, USA).

The baseline, pre-CBT, mid-CBT and post-CBT scores were analyzed between groups with single-factor (time) repeated-measures analyses of variance (ANOVAs) using Greenhouse–Geisser correction. Pairwise differences were measured using paired t-tests with Bonferroni correction to control for Type I error. The adjusted α value was α = .05 / 4 = .013.

The mean changes in our primary outcome measure (LSAS) were calculated among patients showing both symptomatic response and remission. We established the following threshold for response and remission [48]: treatment-responder status was defined as a 31%-or-greater reduction in LSAS score over the course of treatment, and remission was defined as a score of ≤ 36 on the LSAS. Moreover, patients who met the remission criteria were confirmed to no longer meet the criteria for SAD diagnosis using SCID-I interviews conducted by a skilled psychiatrist who was not a CBT therapist.

Moreover, the magnitude of the treatment effect was determined as the effect size ([*M*_*pre* − *CBT*_ − *M*_*post* − *CBT*_]/*SD*_*pre* − *CBT*_) for each scale (LSAS, SPS, SIAS, FNE, and FQ-SP). According to Cohen [49], effect sizes are categorized as follows: small (.20–.49), medium (.50–.79), and large (≥ .80). Effect sizes reported in previous studies were calculated by different methods for various outcome measures. For a direct comparison among different CBT studies, we recalculated the effect sizes for each study based on these measures of SAD severity using the formula [(*M*_*pre* − *CBT*_ − *M*_*post* − *CBT*_)/*SD*_*pre* − *CBT*_].

Finally, in order to elucidate the baseline predictors of treatment outcomes, multiple regression analyses were conducted with post-treatment LSAS scores as a dependent variable and the baseline demographic and clinical variables (gender, age, SAD subtype, presence of comorbid major depressive disorder, presence of another comorbid anxiety disorder, presence of avoidant personality disorder, age of onset, duration of SAD, employment status, marital status, educational status, use of medication, presence of resistance to antidepressants) as independent variables while controlling for baseline LSAS scores.

## Results

### Treatment acceptability by the therapists

All of the participating therapists participated in the CBT training program (Chiba Improving Access for Psychological Therapies project) for 2 years and were able to adhere to the treatment protocol under weekly supervision. The mean CTS-R rating (adjusted for caseload) was 36.1 (on the basis of 15 randomly selected sessions; average SD = .39), which is greater than the threshold of competence expected in UK CBT training programs [40].

### Baseline data

**Figure 1:**
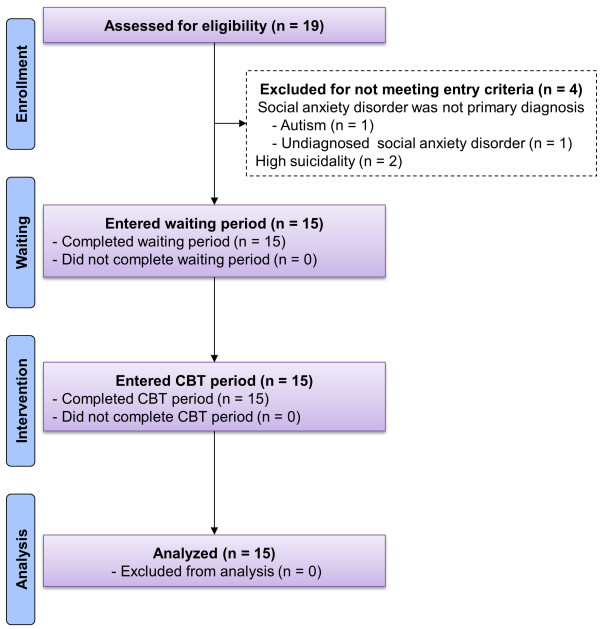
**CONSORT participants’ flow diagram.** Abbreviations: LSAS, Liebowitz Social Anxiety Scale; CBT, Cognitive Behavioral Therapy.

Participants were recruited according to the Consolidated Standards of Reporting Trials (CONSORT) guidelines, as presented in Figure 1. Of the 19 subjects screened, 15 were eligible for the study criteria and referred to the study. After enrolling in the study, no patient dropped out throughout the study. Table 1 shows the baseline demographic and clinical variables of the 15 patients enrolled in this study. The participants included 12 women (80%), and the patients’ mean age was 29.9 years. All participants met the principal DSM-IV diagnostic criteria for SAD. Additional Axis I diagnoses for the patients included major depressive disorder (53%) and other anxiety disorders (e.g., panic disorder with agoraphobia; 7%). Other demographic and clinical variables of the participants are shown in Table 1.

**Table 1:** Baseline demographic and clinical characteristics (N = 15)

**Variable**		**Value**
Gender, female, N (%)		12 (80)
Age, years, Mean (SD)		29.9 (9.2)
Subtype, generalized, N (%)		13 (87)
Comorbid axis I diagnosis, N (%)	No comorbid condition (SAD only)	9(60)
	Mood disorder (major depression)	5 (33)
	Other anxiety disorder (panic)	1 (7)
Avoidant personality disorder, N (%)		4 (27)
Age of onset, years, Mean (SD)		17.6 (8.3)
Duration of SAD, years, Mean (SD)		12.5 (9.8)
Employment status, N (%)	Employed full-time	3 (20)
	Full-time student	6 (40)
	Part-time/homemaker	2 (13)
	Unemployed	4 (26)
Marital status, N (%)	Single	11 (73)
	Married	3 (20)
	Divorced	1 (7)
Educational background, N (%)	Junior high school	2 (13)
	High school	7 (44)
	<3 years of college/university	3 (20)
	≥3 years of college/university	3 (20)
Length of education, years, Mean (SD)		13.7 (1.8)
Current medication, N (%)	Benzodiazepines	5 (33)
	Antidepressants	5 (33)
	Both BZ and AD	3 (20)
	No medication	2 (13)
Resistance to antidepressants, N (%)^1^		11 (73)

^1^: failure to respond to antidepressants: at least one SSRI was inadequate despite maximum dose and treatment duration of at least 12 weeks.

Abbreviations: SAD, Social Anxiety Disorder; BZ, Benzodiazepines; AD, Antidepressants.

### Treatment outcomes

Table 2 presents the mean baseline, pre-CBT, mid-CBT, and post-CBT raw scores for the primary and secondary outcome measures. Single-factor repeated-measures ANOVAs showed significant main effects of time on all outcome measures after the completion of treatment (*p* < .001). Pairwise comparisons of outcome measures indicated that the completers did not improve on any measure during the 2-week waiting period (baseline to pre-CBT); this indicates the baseline stability of their symptoms. On the other hand, the CBT intervention led to significant reductions in all outcome measures at the middle stage of treatment (pre–mid-CBT; *p* < .05) and a further significant reduction after treatment completion (mid–post-CBT; *p* < .05). On the basis of our primary outcome measure (LSAS), 11 patients (73.3%) were judged to be responders, and 6 of them (40%) met the criteria for SAD remission at the post-CBT evaluation.

**Table 2:** Repeated-measures analyses of variance (N = 15)

	**Baseline**	**Pre-CBT**	**Mid-CBT**	**Post-CBT**		
	**Mean (SD)**	**Mean (SD)**	**Mean (SD)**	**Mean (SD)**	**Time ( *F *)**	**η**^**2**^
**LSAS**	90.5 (21.4)^a^	91.8 (23.5)^a^	72.3 (21.5)^b^	51.7 (27.8)^c^	27.3^***^	.66
**SPS**	42.4 (17.3)^a^	44.1 (18.7)^a^	32.8 (14.5)^b^	26.1 (17.6)^c^	18.9^***^	.57
**SIAS**	57.7 (11.9)^a^	58.7 (12.0)^a^	47.7 (13.6)^b^	37.1 (19.0)^c^	33.3^***^	.70
**FNE**	25.4 (3.9)^a^	25.2 (4.6)^a^	21.1 (6.2)^b^	16.9 (9.1)^c^	16.2^***^	.54
**FQ-SP**	25.4 (8.1)^a^	28.5 (8.2)^a^	19.9 (9.5)^b^	16.0 (10.1)^c^	15.9^***^	.54

^***^ Significant effect of time (*p* < .001).

Note: The same letters are not significantly different in pairwise comparisons.

Abbreviations: LSAS, Liebowitz Social Anxiety Scale; SPS, Social Phobia Scale; SIAS, Social Interaction Anxiety Scale; FNE, Fear of Negative Evaluation Scale; FQ-SP, Fear Questionnaire – Social Phobia subscale.

As shown in Table 3, our pre–post-CBT effect sizes were large and provided comparable effectiveness to those obtained in previous studies of individual CBT, as determined using the model of Clark and Wells for all social-severity scales (effect sizes for LSAS = 1.71, SPS = 0.96, SIAS = 1.80, FNE = 1.82 and FQ-SP = 1.54).

**Table 3:** Comparison of effect sizes among various clinical trials using the model of Clark and Wells

**Study group**		**Present study**			**Clark et al. (2003)**			**Stangier et al. (2003)**			**Clark et al. (2006)**			**Mörtberg et al. (2007)**			**Stangier et al. (2011)**		
**CBT protocol**		**90 min 14 weeks**			**75 min 16 weeks**			**60 min 15 weeks**			**90 min0 14 weeks**			**60 min 16 weeks**			**50–90 min 20 weeks**		
		**Pre**	**Post**	**ES**	**Pre**	**Post**	**ES**	**Pre**	**Post**	**ES**	**Pre**	**Post**	**ES**	**Pre**	**Post**	**ES**	**Pre**	**Post**	**ES**
LSAS	Mean (SD)	91.8 (23.5)	51.7 (27.8)	1.71	78.7 (25.6)	35.4 (22.9)	1.69				74.8 (24.1)	28.0 (17.7)	1.94	81.8 (21.1)	51.3 (27.9)	1.45	69.2 (23.4)	39.5 (21.1)	1.27
SPS	Mean (SD)	44.1 (18.7)	26.1 (17.6)	0.96	30.2 (14.8)	17.4 (13.2)	0.86	30.7 (10.2)	21.5 (12.6)	0.90	29.3 (13.5)	9.0 (6.0)	1.51	37.9 (12.5)	21.5 (13.8)	1.31			
SIAS	Mean (SD)	58.7 (12.0)	37.1 (19.0)	1.80	48.3 (12.3)	34 (13.9)	1.17	44.9 (10.8)	36.1 (14.8)	0.82	43.6 (17.8)	18.2 (10.0)	1.43	51.6 (15.5)	37.8 (17.7)	0.89			
FNE	Mean (SD)	25.2 (4.6)	16.9 (9.1)	1.82	25.2 (5.2)	19.5 (8.7)	1.09				23.1 (7.0)	12.9 (8.9)	1.46	23.3 (4.8)	18.5 (7.4)	1.00			
FQ	Mean (SD)	28.5 (8.2)	16.5 (10.7)	1.54	22.4 (6.4)	14.2 (7.0)	1.29							22.3 (8.6)	14.6 (7.6)	0.9			

Abbreviations: LSAS, Liebowitz Social Anxiety Scale; SPS, Social Phobia Scale; SIAS, Social Interaction Anxiety Scale; FNE, Fear of Negative Evaluation; FQ-SP, Fear Questionnaire – Social Phobia subscale; ES, Effect Size.

### Predictors of CBT outcomes

None of the baseline demographic and clinical variables (gender, age, SAD subtype, presence of comorbid major depressive disorder, presence of another comorbid anxiety disorder, presence of avoidant personality disorder, age of onset, duration of SAD, employment status, marital status, educational status, use of medication, and presence of resistance to antidepressants) were significant predictors of post-treatment LSAS score.

## Discussion

This single-arm trial demonstrated that individual CBT, which was originally developed in Western countries, could lead to a significant reduction in SAD severity among Japanese patients. Moreover, our individual CBT demonstrated excellent acceptability, considering that there was no dropout among the participants.

Although the severity of SAD among our recruited patients was higher than that observed in previous studies (see Table 3), our effect size of 1.71 in terms of LSAS scores between the pre- and post-CBT observations is comparable to the effect sizes of 1.29–1.94 reported in previous clinical trials on CBT that used the model of Clark and Wells [28,29,37-39]. Comparison of effect sizes among various studies may be difficult, because the LSAS was administered by different methods (self-reported vs. clinician-administered) across the different studies. However, it is thought to be possible to compare these different types of LSAS scores, because scores on the self-report version of the LSAS correspond well to those on the clinician-administered version: In a previous study group, there was little difference between the two versions of the LSAS on any scale or subscale score [50].

Our identification of possible predictors of response to CBT showed that the observed baseline demographic and clinical variables were not statistically significant predictors of LSAS scores after receiving CBT. The presence of comorbid mood disorders did not predict CBT outcomes in this study, and some previous studies have also demonstrated that individuals with comorbid mood disorders responded similarly to those with uncomplicated SAD [51,52]. However, Blanco (2003) found pretreatment levels of self-reported depression to be the single most significant predictor of treatment outcomes [53]. Thus, it may be necessary to assess the level of severity of comorbid depression to facilitate the prediction of CBT outcomes in further studies. As for antidepressant history, it is reported that about 50% of individuals do not respond to antidepressants or have residual symptoms after first-line antidepressant treatment [54]. Most patients (73%) who participated in the current study showed resistance to first-line medications (at least one course of SSRI administration at the maximum dose for at least 12 weeks). One reason for this pattern of results is that only SSRIs (fluvoxamine and paroxetine) have been approved as treatments for SAD in Japan as of 2012. However, in the present study, CBT decreased SAD severity similarly in antidepressant-resistant patients and antidepressant-responsive ones (i.e., the presence of antidepressant resistance was not a predictor of CBT outcomes). This finding suggested that individual CBT might have potential as a next-step strategy even for cases of antidepressant-resistant SAD.

Though the present study provided valuable information, its design imposes the following limitations. (1) This study included only a small sample size, which resulted in limited generalizability of its conclusions. (2) This was a single-center study; therefore, our participating population was somewhat different from those seen in routine clinical practice. Our study was designed to recruit patients similar to those seen in routine clinical practice; as a result, 40% had comorbid disorders, as is typical in clinical practice [55]. However, although the gender ratio of SAD cases is not typically skewed, the participants in this study were mostly women. The reason for this was that our outpatient section always took appointments during the daytime on weekdays; therefore, many men who worked weekdays could not participate in this study. (3) The lack of follow-up data limits the generalizability of the study’s conclusions to longer-term outcomes. This is an important consideration in evaluating the effectiveness of CBT, because CBT has unknown characteristics in terms of longer-term effects, associated relapse rates, cost-effectiveness characteristics, etc. (4) Psychotropic medication intake could not be discontinued before the start of this study. Though the inclusion of subjects using psychotropic medication limits the generalizability of the results, most patients (73%) already showed resistance to first-line medications (such as SSRIs) at baseline, and all of the patients showed baseline stability of their symptoms during the 2-week waiting period. Thus, it seemed that our individual CBT did indeed reduce SAD severity. (5) This was an uncontrolled study; therefore, we could not conclude definitively that our individual CBT was effective. It remains unknown whether the observed improvement in SAD severity is related to the natural course of SAD.

Future study should replicate these findings and address the limitations of this study in multi-center, randomized, controlled trials conducted with larger and more diverse samples across longer follow-up periods.

## Conclusions

Despite several limitations, this study suggests via a single-arm design that individual CBT is a feasible treatment, even for Japanese patients with SAD. Further controlled trials that address the limitations of this study are required.

### Availability of supporting data

The data sets supporting the results of this article are included within the article.

### Consent

Written informed consent was obtained from the patient for publication of this report and any accompanying images.

## Abbreviations

AD: Antidepressants; BZ: Benzodiazepines; CBT: Cognitive behavior therapy; ES: Effect size; FNE: Fear of negative evaluation; FQ-SP: Fear questionnaire – social phobia subscale; LSAS: Liebowitz social anxiety scale; SAD: Social anxiety disorder; SIAS: Social interaction anxiety scale; SSRI: Selective serotonin reuptake inhibitor; SPS: Social phobia scale

## Competing interests

The authors declare that they have no competing interests.

## Authors’ contributions

NY designed and managed the study, performed the statistical analyses, and drafted the manuscript. FO, SM, MT, HI, and TK performed the clinical treatment and management. KA, OK, TS, EI, AN, MN, and MI participated in the design of the study conception, administered supervised therapies, and coordinated the trial. ES administered supervised therapies, performed clinical investigation (diagnosis) and supervised the overall conduct of the study. All authors read, critically revised, and approved the final manuscript.

## References

[B1] American Psychiatric Association Diagnostic and Statistical Manual of Mental Disorders, Fourth Edition: DSM-IV-TR 2000 Washington: American Psychiatric Pub

[B2] Stein DJ Ruscio AM Lee S Petukhova M Alonso J Andrade LHSG Benjet C Bromet E Demyttenaere K Florescu S de Girolamo G de Graaf R Gureje O He Y Hinkov H Hu C Iwata N Karam EG Lepine J-P Matschinger H Oakley Browne M Posada-Villa J Sagar R Williams DR Kessler RC Subtyping social anxiety disorder in developed and developing countries Depress Anxiety 2010 27 390 403 10.1002/da.20639 20037919 PMC2851829

[B3] Stein MB Roy-Byrne PP Craske MG Bystritsky A Sullivan G Pyne JM Katon W Sherbourne CD Functional impact and health utility of anxiety disorders in primary care outpatients Med Care 2005 43 1164 1170 10.1097/01.mlr.0000185750.18119.fd 16299426

[B4] Wang PS Lane M Olfson M Pincus HA Wells KB Kessler RC Twelve-month use of mental health services in the United States: results from the National Comorbidity Survey Replication Arch Gen Psychiatry 2005 62 629 640 10.1001/archpsyc.62.6.629 15939840

[B5] Sareen J Jacobi F Cox BJ Belik S-L Clara I Stein MB Disability and poor quality of life associated with comorbid anxiety disorders and physical conditions Arch Intern Med 2006 166 2109 2116 10.1001/archinte.166.19.2109 17060541

[B6] Bandelow B Zohar J Hollander E Kasper S Möller H-J Zohar J Hollander E Kasper S Möller H-J Bandelow B Allgulander C Ayuso-Gutierrez J Baldwin DS Buenvicius R Cassano G Fineberg N Gabriels L Hindmarch I Kaiya H Klein DF Lader M Lecrubier Y Lépine J-P Liebowitz MR Lopez-Ibor JJ Marazziti D Miguel EC Oh KS Preter M Rupprecht R World Federation of Societies of Biological Psychiatry (WFSBP) guidelines for the pharmacological treatment of anxiety, obsessive-compulsive and post-traumatic stress disorders - first revision World J Biol Psychiatry 2008 9 248 312 10.1080/15622970802465807 18949648

[B7] Stein DJ Baldwin DS Bandelow B Blanco C Fontenelle LF Lee S Matsunaga H Osser D Stein MB van Ameringen M A 2010 evidence-based algorithm for the pharmacotherapy of social anxiety disorder Curr Psychiatry Rep 2010 12 471 477 10.1007/s11920-010-0140-8 20686872

[B8] Blanco C Bragdon LB Schneier FR Liebowitz MR The evidence-based pharmacotherapy of social anxiety disorder Int J Neuropsychopharmacol 2013 16 235 249 10.1017/S1461145712000119 22436306

[B9] Hofmann SG Smits JAJ Cognitive-behavioral therapy for adult anxiety disorders: a meta-analysis of randomized placebo-controlled trials J Clin Psychiatry 2008 69 621 632 10.4088/JCP.v69n0415 18363421 PMC2409267

[B10] Blomhoff S Haug TT Hellström K Holme I Humble M Madsbu HP Wold JE Randomised controlled general practice trial of sertraline, exposure therapy and combined treatment in generalised social phobia Br J Psychiatry 2001 179 23 30 10.1192/bjp.179.1.23 11435264

[B11] Davidson JRT Foa EB Huppert JD Keefe FJ Franklin ME Compton JS Zhao N Connor KM Lynch TR Gadde KM Fluoxetine, comprehensive cognitive behavioral therapy, and placebo in generalized social phobia Arch Gen Psychiatry 2004 61 1005 1013 10.1001/archpsyc.61.10.1005 15466674

[B12] Gelernter CS Uhde TW Cimbolic P Arnkoff DB Vittone BJ Tancer ME Bartko JJ Cognitive-behavioral and pharmacological treatments of social phobia. A controlled study Arch Gen Psychiatry 1991 48 938 945 10.1001/archpsyc.1991.01810340070009 1929764

[B13] Heimberg RG Liebowitz MR Hope DA Schneier FR Holt CS Welkowitz LA Juster HR Campeas R Bruch MA Cloitre M Fallon B Klein DF Cognitive behavioral group therapy vs phenelzine therapy for social phobia: 12-week outcome Arch Gen Psychiatry 1998 55 1133 1141 10.1001/archpsyc.55.12.1133 9862558

[B14] Hofmann SG Barlow DH Papp LA Detweiler MF Ray SE Shear MK Woods SW Gorman JM Pretreatment attrition in a comparative treatment outcome study on panic disorder Am J Psychiatry 1998 155 43 47 9433337 10.1176/ajp.155.1.43

[B15] Lepola U Bergtholdt B Lambert B St Davy KL Ruggiero L Controlled-release paroxetine in the treatment of patients with social anxiety disorder J Clin Psychiatry 2004 65 222 229 10.4088/JCP.v65n0213 15003077

[B16] Liebowitz MR Gelenberg AJ Munjack D Venlafaxine extended release vs placebo and paroxetine in social anxiety disorder Arch Gen Psychiatry 2005 62 190 198 10.1001/archpsyc.62.2.190 15699296

[B17] Ono Y Furukawa TA Shimizu E Okamoto Y Nakagawa A Fujisawa D Nakagawa A Ishii T Nakajima S Current status of research on cognitive therapy/cognitive behavior therapy in Japan Psychiatry Clin Neurosci 2011 65 121 129 10.1111/j.1440-1819.2010.02182.x 21414087

[B18] Prince R Tcheng-Laroche F Culture-bound syndromes and international disease classifications Cult Med Psychiatry 1987 11 3 52 10.1007/BF00055003 3829693

[B19] Russell JG Anxiety disorders in Japan: a review of the Japanese literature on shinkeishitsu and taijinkyofusho Cult Med Psychiatry 1989 13 391 403 10.1007/BF00052047 2612189

[B20] Kleinknecht RA Dinnel DL Kleinknecht EE Hiruma N Harada N Cultural factors in social anxiety: a comparison of social phobia symptoms and Taijin kyofusho J Anxiety Disord 1997 11 157 177 10.1016/S0887-6185(97)00004-2 9168340

[B21] Suzuki K Takei N Kawai M Minabe Y Mori N Is taijin kyofusho a culture-bound syndrome? Am J Psychiatry 2003 160 1358 10.1176/appi.ajp.160.7.1358 12832264

[B22] Choy Y Schneier FR Heimberg RG Oh K-S Liebowitz MR Features of the offensive subtype of Taijin-Kyofu-Sho in US and Korean patients with DSM-IV social anxiety disorder Depress Anxiety 2008 25 230 240 10.1002/da.20295 17340609

[B23] Hofmann SG Anu Asnaani MA Hinton DE Cultural aspects in social anxiety and social anxiety disorder Depress Anxiety 2010 27 1117 1127 10.1002/da.20759 21132847 PMC3075954

[B24] Stein DJ Social anxiety disorder in the West and in the East Ann Clin Psychiatry 2009 21 109 117 19439161

[B25] Chen J Nakano Y Ietzugu T Ogawa S Funayama T Watanabe N Noda Y Furukawa TA Group cognitive behavior therapy for Japanese patients with social anxiety disorder: preliminary outcomes and their predictors BMC Psychiatry 2007 7 69 10.1186/1471-244X-7-69 18067685 PMC2241595

[B26] Chen J Furukawa TA Nakano Y Ietsugu T Ogawa S Funayama T Watanabe N Noda Y Rapee RM Video feedback with peer ratings in naturalistic anxiety-provoking situations for social anxiety disorder: preliminary report J Behav Ther Exp Psychiatry 2010 41 6 10 10.1016/j.jbtep.2009.08.005 19729149

[B27] Watanabe N Furukawa TA Chen J Kinoshita Y Nakano Y Ogawa S Funayama T Ietsugu T Noda Y Change in quality of life and their predictors in the long-term follow-up after group cognitive behavioral therapy for social anxiety disorder: a prospective cohort study BMC Psychiatry 2010 10 81 10.1186/1471-244X-10-81 20942980 PMC2965130

[B28] Mörtberg E Clark DM Sundin O Aberg Wistedt A Intensive group cognitive treatment and individual cognitive therapy vs. treatment as usual in social phobia: a randomized controlled trial Acta Psychiatr Scand 2007 115 142 154 10.1111/j.1600-0447.2006.00839.x 17244178

[B29] Stangier U Heidenreich T Peitz M Lauterbach W Clark DM Cognitive therapy for social phobia: individual versus group treatment Behav Res Ther 2003 41 991 1007 10.1016/S0005-7967(02)00176-6 12914803

[B30] Liebowitz MR Social phobia Mod Probl Pharmacopsychiatry 1987 22 141 173 2885745 10.1159/000414022

[B31] Raj BA Sheehan DV Social anxiety disorder Med Clin North Am 2001 85 711 733 10.1016/S0025-7125(05)70337-0 11349481

[B32] Sugawara N Yasui-Furukori N Kaneda A Sato Y Tsuchimine S Fujii A Danjo K Takahashi I Matsuzaka M Kaneko S Factor structure of the Liebowitz Social Anxiety Scale in community-dwelling subjects in Japan Psychiatry Clin Neurosci 2012 66 525 528 10.1111/j.1440-1819.2012.02381.x 22988811

[B33] Baron-Cohen S Wheelwright S Skinner R Martin J Clubley E The autism-spectrum quotient (AQ): evidence from Asperger syndrome/high-functioning autism, males and females, scientists and mathematicians J Autism Dev Disord 2001 31 5 17 10.1023/A:1005653411471 11439754

[B34] First MB Gibbon M User’s Guide for the Structured Clinical Interview for DSM-IV Axis I Disorders SCID-I: Clinician Version 1997 Washington: American Psychiatric Pub

[B35] First MB Gibbon M User’s Guide for the Structured Clinical Interview for DSM-IV Axis II Personality Disorders: SCID-II 1997 Washington: American Psychiatric Pub

[B36] Clark DM Wells A Heimberg RG, Liebowitz M, Hope DA, Schneier FR A cognitive model of social phobia Social Phobia: Diagnosis, Assessment, and Treatment 1995 New York: Guilford Press 69 93

[B37] Clark DM Ehlers A McManus F Hackmann A Fennell M Campbell H Flower T Davenport C Louis B Cognitive therapy versus fluoxetine in generalized social phobia: a randomized placebo-controlled trial J Consult Clin Psychol 2003 71 1058 1067 14622081 10.1037/0022-006X.71.6.1058

[B38] Clark DM Ehlers A Hackmann A McManus F Fennell M Grey N Waddington L Wild J Cognitive therapy versus exposure and applied relaxation in social phobia: a randomized controlled trial J Consult Clin Psychol 2006 74 568 578 16822113 10.1037/0022-006X.74.3.568

[B39] Stangier U Schramm E Heidenreich T Berger M Clark DM Cognitive therapy vs interpersonal psychotherapy in social anxiety disorder: a randomized controlled trial Arch Gen Psychiatry 2011 68 692 700 10.1001/archgenpsychiatry.2011.67 21727253

[B40] Blackburn I-M James IA Milne DL Baker C Standart S Garland A Reichelt FK The Revised Cognitive Therapy Scale (CTS-R): psychometric properties Behav Cogn Psychother 2001 29 431 446

[B41] Mattick RP Clarke JC Development and validation of measures of social phobia scrutiny fear and social interaction anxiety Behav Res Ther 1998 36 455 470 10.1016/S0005-7967(97)10031-6 9670605

[B42] Watson D Friend R Measurement of social-evaluative anxiety J Consult Clin Psychol 1969 33 448 457 5810590 10.1037/h0027806

[B43] Marks IM Mathews AM Brief standard self-rating for phobic patients Behav Res Ther 1979 17 263 267 10.1016/0005-7967(79)90041-X 526242

[B44] Asakura S Inoue S Sasaki F Sasaki Y Kitagawa N Inoue T Denda K Ito M Matsubara R Koyama T Reliability and validity of the Japanese version of the Liebowitz Social Anxiety Scale Seishin Igaku (Clinical Psychiatry) 2002 44 1077 1084

[B45] Kanai Y Sasakawa S Chen J Suzuki S Shimada H Sakano Y Development and validation of the Japanese version of Social Phobia Scale and Social Interaction Anxiety Scale Shingshin-Igaku (Jpn J Psychosom Med) 2004 44 841 850

[B46] Ishikawa R Sasaki K Fukui I Standardization of Japanese version of FNE and SADS Koudou Ryouhou Kenkyu (Jap J Behav Ther) 1992 18 10 17

[B47] Mohr DC Spring B Freedland KE Beckner V Arean P Hollon SD Ockene J Kaplan R The selection and design of control conditions for randomized controlled trials of psychological interventions Psychother Psychosom 2009 78 275 284 10.1159/000228248 19602916

[B48] Bandelow B Baldwin DS Dolberg OT Andersen HF Stein DJ What is the threshold for symptomatic response and remission for major depressive disorder, panic disorder, social anxiety disorder, and generalized anxiety disorder? J Clin Psychiatry 2006 67 1428 1434 10.4088/JCP.v67n0914 17017830

[B49] Cohen J Statistical Power Analysis in the Behavioral Sciences 1988 2 Hillsdale: Erlbaum

[B50] Fresco DM Coles ME Heimberg RG Liebowitz MR Hami S Stein MB Goetz D The Liebowitz Social Anxiety Scale: a comparison of the psychometric properties of self-report and clinician-administered formats Psychol Med 2001 31 1025 1035 11513370 10.1017/s0033291701004056

[B51] Marom S Gilboa-Schechtman E Aderka IM Weizman A Hermesh H Impact of depression on treatment effectiveness and gains maintenance in social phobia: a naturalistic study of cognitive behavior group therapy Depress Anxiety 2009 26 289 300 10.1002/da.20390 19170088

[B52] Chambless DL Tran GQ Glass CR Predictors of response to cognitive-behavioral group therapy for social phobia J Anxiety Disord 1997 11 221 240 10.1016/S0887-6185(97)00008-X 9220298

[B53] Blanco C Schneier FR Schmidt A Blanco-Jerez C-R Marshall RD Sánchez-Lacay A Liebowitz MR Pharmacological treatment of social anxiety disorder: a meta-analysis Depress Anxiety 2003 18 29 40 10.1002/da.10096 12900950

[B54] Van Ameringen M Mancini C Pipe B Bennett M Optimizing treatment in social phobia: a review of treatment resistance CNS Spectr 2004 9 753 762 15448580 10.1017/s1092852900022392

[B55] Stein MB Stein DJ Social anxiety disorder Lancet 2008 371 1115 1125 10.1016/S0140-6736(08)60488-2 18374843

